# Emotional distress and adjustment in patients with end-stage kidney disease: A qualitative exploration of patient experience in four hospital trusts in the West Midlands, UK

**DOI:** 10.1371/journal.pone.0241629

**Published:** 2020-11-05

**Authors:** Kim Sein, Sarah Damery, Jyoti Baharani, Johann Nicholas, Gill Combes

**Affiliations:** 1 Institute of Applied Health Research, University of Birmingham, Birmingham, West Midlands, United Kingdom; 2 Renal Unit, Birmingham Heartlands Hospital, Birmingham, West Midlands, United Kingdom; 3 Renal Unit, Royal Shrewsbury Hospital, Shrewsbury, Shropshire, United Kingdom; University of Missippi Medical Center, UNITED STATES

## Abstract

**Objectives:**

To explore patient perceptions and experiences of mild-to-moderate emotional distress and the support offered by kidney units to patients with end-stage kidney disease.

**Methods:**

In-depth, semi-structured qualitative interviews with patients (n = 46) being treated for end-stage kidney disease in four hospital Trusts, with data analysed thematically.

**Results:**

Patients described multiple sources of distress and talked about the substantial burden that emotional challenges raised for their ability to manage their condition and develop coping strategies. Many patients did not feel it appropriate to disclose their emotional issues to staff on the kidney unit, due to a perceived lack of time for staff to deal with such issues, or a perception that staff lacked the necessary skills to provide resolution. Five themes were identified from the patient interviews, broadly related to patients’ experience of distress, and the support offered by the kidney unit: i) the emotional burden that distress placed on patients; ii) patients’ relationship with the treatment for their condition; iii) strategies for coping and adjustment; iv) patient-staff interactions and the support offered by the kidney unit, and v) the mediating impact of the treatment environment on patient experience of distress and their ability to raise emotional issues with staff.

**Conclusions:**

Many patients felt unprepared for the likelihood of experiencing emotional issues as part of their condition, for which pre-dialysis education could help in managing expectations, along with support to help patients to develop appropriate coping strategies and adjustments. These findings demonstrate the importance of recognising patient distress and ensuring that talking about distress becomes normalised for patients with end-stage kidney disease.

## Introduction

At the end of 2017, 64,887 patients with end-stage kidney disease (ESKD) were receiving kidney replacement therapy in England [1]. ESKD is a chronic condition in which treatment is life-saving but not curative [2]. Patients with ESKD face numerous challenges related to their health status and ongoing treatment that introduces substantial uncertainty into their lives [3]. Consequently, patients can experience a plethora of emotional and psychological problems [4]. The prevalence of depression and anxiety in patients with chronic conditions is around 1.5 to 4 times greater than in the general population [5, 6], and anxiety is among the top patient and caregiver priorities for outcomes in Chronic Kidney Disease [7]. Approximately 20–30% of dialysis patients [8], and 25% of transplant patients [9] experience depression and anxiety. Similar ranges have been shown in people with other long-term conditions such as type two diabetes (18 to 35%) [10]; and Chronic Obstructive Pulmonary Disease (COPD) (20%) [11]. Results from the cross-sectional survey element of this study showed that 33.3% of people with ESKD met criteria for mild to moderate distress [12]. Similarly, a recent analysis of haemodialysis patients [13] showed 39% of patients demonstrated depression using the validated Patient-Health Questionnaire-9 [14].

Appropriate support for patients who experience emotional and psychological problems is central to UK policy on managing long-term conditions [15] and kidney disease [16]. Both the Department of Health (DH) and National Institute for Health and Care Excellence (NICE) mandate the provision of emotional and psychological support within their national kidney guidelines [17, 18], and patients with ESKD also support embedding effective treatment of emotional difficulties within kidney units [19]. However, patient support is generally targeted towards patients requiring psychological or psychiatric intervention, and lower-level needs (‘distress’) in kidney patients may remain unrecognised or untreated [20]. This is particularly problematic given that lower-level problems may be associated with poorer outcomes [21, 22].

Definitions of distress originate in the field of oncology, where distress has been argued to “extend along a continuum ranging from common normal feelings of vulnerability, sadness and fears, to problems that can become disabling such as depression, anxiety, panic and social isolation” [23]. Whilst multiple studies have explored patient experience of anxiety and depression within the context of kidney failure [24], there is little research describing patients’ experience of living with distress and undergoing treatment for ESKD. This study used semi-structured interviews to explore patients’ experience of mild-to-moderate distress in ESKD.

## Materials and methods

This study was approved by the NRES West Midlands Coventry and Warwickshire Research Ethics Committee (Ref: 15/WM/0288) and the Health Research Authority (IRAS Ref: 184996). Approval was also obtained from the Research Governance office of each of the participating National Health Service (NHS) Trusts.

The detailed study methodology is available in the published protocol [25]. Briefly, semi-structured, in-depth qualitative interviews were undertaken with clinically stable adult patients (aged 18 and over) being treated at four kidney units with diverse patient populations located in urban areas in the West Midlands, England. Eligible patients were those receiving dialysis treatment (in-centre haemodialysis–HD; home haemodialysis–HHD, peritoneal dialysis–PD); patients who were yet to start treatment, and patients with a kidney transplant.

Patients were recruited following a survey which used the distress and emotion thermometers [26] to identify respondents who met the criteria for mild-to-moderate distress i.e. scores of between 4 and 7 on the distress thermometer (DT) or between 0 and 3 on the DT and between 4 and 7 on one or more of the four domains measured by the emotion thermometers (anxiety, depression, anger, perceived need for help). Patients meeting these criteria, who also indicated willingness to be interviewed and provided contact details in their survey return were purposively sampled in order to achieve maximum variation by age, sex, ethnic group and treatment type.

Eligible patients were telephoned by a member of the research team to confirm their willingness to be interviewed and to record their preference for a face-to-face or telephone interview. Patients were then posted a consent form and Participant Information Sheet (PIS) which explained the purpose of the interview and what participation would involve. A further telephone call was made a week later to confirm participation and arrange an interview time. Telephone (n = 43) or face-to-face interviews in the patient’s home (n = 3) were undertaken between April 2016 and May 2017 by two female qualitative academic researchers (FT, EK), who were qualified to Masters level and employed by the University of Birmingham. The interviewers had no personal experience of, or particular personal interest in the research topic. Written consent was obtained from all participants.

All interviews lasted between 25 and 55 minutes and involved one interviewer and one interviewee at a time. Interviews were structured around a topic guide informed by the literature, kidney patients with an advisory role on the study, and clinicians and academics on the project advisory group. The topic guide was designed to explore issues related to distress, coping, adjustment and support [25]. Patients were asked about their experience of distress and the support they felt they needed, staff recognition of distress and support offered, and whether they could make any recommendations about how kidney units could improve the recognition of, and response to patients’ emotional challenges. In the case of participants undergoing in-centre HD, interviews were always scheduled to take place on a different day to a planned HD session to avoid the likelihood of post-dialysis fatigue impacting on participants’ emotional perspectives. Data saturation was reached after 46 interviews had been completed. No participants chose to withdraw from the study. Interviews were audio-recorded and professionally transcribed verbatim, with transcripts proofread against the recordings to ensure accuracy. Interviewees were not given the opportunity to check transcripts.

Interview data were analysed thematically [27]. Two researchers (GC, KS) independently coded eight of the 46 patient transcripts. Following comparison and discussion, an initial coding framework was developed, which was used by KS to code the remaining transcripts using NVivo software. During coding, regular discussion of ordinate and subordinate notes led to refinement of the detailed coding structure. Where data did not fit existing themes, new codes were developed or existing ones revised. A random selection of 10% of transcripts was coded independently by GC to check for consistency, with minor amendments subsequently made to the coding framework. Thematic analysis was then used to compare and cluster codes so that emerging themes could be identified. These were discussed by the research team, and the findings were refined in an iterative process.

### Patient and public involvement

The study was reviewed by a Patient and Public Involvement (PPI) advisory group who had previously been involved in other kidney research. Following PPI feedback, changes were made to the topic guide (the addition of questions related to adjustment and refinement of question prompts).

## Results

A total of 46 interviews were undertaken. Table 1 describes key participant characteristics.

**Table 1:** Participant characteristics.

Participant group		Number of interviews
ESKD treatment	Pre-dialysis	8
	Dialysis <2 years	9
	Dialysis 2+ years	15
	Transplant	14
Sex	Male	28
	Female	18
Age group	Under 50 years	10
	50 to 69	22
	70+ years	14
Ethnicity	White	33
	Black, Asian, and minority ethnic	13
Dialysis type (n = 24)	In-centre haemodialysis	19
	Home haemodialysis	4
	Peritoneal dialysis	1
Type of interview	Telephone	43
	Face-to-face	3

Five key themes were identified (Table 2). Three themes focus on patients’ experience of distress and the extent to which patients were able to develop coping strategies; two further themes describe patients’ views about the kidney unit itself, and the support offered by staff.

**Table 2:** Interview themes.

	Themes
Patients’ experience of distress	The emotional burden of distress
	Patients’ relationship with treatment
	Coping and adjustment
The kidney unit and support	Patient-staff interaction and kidney unit support
	Impact of the treatment environment

### The emotional burden of distress

The majority of patients described a substantial emotional burden from their illness. Feelings of helplessness, loss of control and anger were mixed with frustration and distress, which created a difficult emotional state for many:

*“…it’s a hard one to explain because the reality is that I’ve just got to deal with it*. *And what I mean by deal with it is that whether I get angry*, *upset*, *or you know*, *downright rude*, *but the reality is that I have renal failure and I’ve got to be on dialysis three times a week… And I guess the anger is just that I don’t feel like it was my fault*.*”* (Female, 40–49 years, HD)

The dynamic nature of distress was frequently described, with many participants talking about experiencing a ‘rollercoaster’ of emotions, such that their emotional state fluctuated between helplessness and being more in control of their feelings:

*‘There are times when you don’t feel anything*, *you see*, *you are just like a robot*. *And then sometimes you feel that you are human*, *you are living*.*’ (*Male, 50–69 years, HD)

Several participants spoke of a perceived loss of identity when they were diagnosed with kidney failure, and described the challenges that their condition had placed on their ability to live a normal life. For some, this meant that they had needed to give up work because of ill-health, which had impacted on their independence and their perceptions about ‘who they were’:

*‘Any sort of major life change*. *Certainly for me when I left work*, *that was a really big change you know*. . . .*It’s a big part of my identity to have other things besides renal failure*. *So giving up work was a big thing and because I had to give up because my health was getting so bad at the time*.*’* (Female, 50–69 years, transplant)

### Patients’ relationship with treatment

Patients tended to have a difficult relationship with dialysis treatment. The majority appreciated that dialysis was a lifesaving treatment but resented the limiting effects it had on their life. For example, medication side-effects were often described as unpleasant, and nearly all participants described dialysis-induced fatigue. For participants receiving in-centre haemodialysis, the need to undertake and prepare for dialysis sessions meant that these sessions were never far from their thoughts and placed substantial restrictions on their lives:

*“Everything has to be planned around that particular day*. *If I want to go down to my daughter’s place for the weekend*, *I’ve got to be back by six o’clock on Monday morning… So you’re restricted*, *your life is ruled by the three appointments per week*. *Even if I’ve got another appointment anywhere*, *it can’t be done on a Monday*, *Wednesday*, *or Friday*. *So that sort of throws half the week out*.*”* (Male, 70+ years, HD)

Many patients described a paradoxical relationship with dialysis, in which they often felt frustrated, upset and trapped by their treatment regime and the restrictions it entailed, whilst at the same time being grateful for the life-saving treatment, without which their life expectancy would be severely curtailed. Dialysis was often described as being like a job, particularly when the often-complicated medication regime and associated fluid and dietary restrictions were considered:

*“I shouldn’t really be here and I’m very appreciative of that… I think people struggle to know what to say*. *I don’t like people feeling sorry for me*, *but I also like people to appreciate that life is a bit harder when you*…*it’s like having a part-time job doing the routine*, *monitoring all the medication*, *organising the stock*, *all the hospital appointments*.*”* (Female, 50–69 years, PD)

Some patients felt that they had not been given enough information about what their treatment would entail or how it would make them feel. This caused anxiety and concern, particularly for those who felt that they had not been involved enough in decisions about their treatment. This led to a perceived loss of control over their illness and its management:

*‘I don’t know what I’m supposed to know and what I’m not supposed to know*. *Once I came out of hospital it was like I’d just been left to get on with it and I don’t feel that’s how I want to be treated*. *I want to understand what my treatment is*. *I want to have a say in how I’m treated and that’s not quite happened to me personally*.*’* (Female, 40–49 years, HD)

Patients also frequently described frustration that the emotional impact of treatment had not been discussed with them before their treatment started. One participant in particular identified a number of important transitional points at which information about their condition, its progression, and the likely emotional burden of this would have been helpful:

*‘I think particularly at the big milestone events*, *whether it’s being told that you have a condition that’s going to end up in renal failure*, *at the point you’ve gone into renal failure*, *at the point you’re going to have treatment like dialysis or when you have a transplant*. *It’s a big transition at that stage that I think knowing or understanding the emotional impact would be more*. . . . *I think would be useful*.*’* (Male, 50–69 years, transplant)

### Coping and adjustment

Few patients described having developed coping strategies to deal with their emotional challenges. Those that did feel better able to cope with their illness tended to describe a close relationship with particular staff within the kidney unit, having a strong support network of family and friends, and feeling in control of their condition and its treatment:

*‘Knowing you’ve got a good team of people behind you*. *When I go to the hospital it’s not only the transplant team*, *it’s also I see regular nurses*…*I’ve seen the same faces for years now*…*And I think they tend to*, *they’re your little rocks to lean on*. *And that helps a lot*.*’* (Male, 70+ years, transplant)

One patient talked about adopting a practical strategy for adjusting to her inability to do many of the things she had done before her ESKD diagnosis:

‘*Adapting my regime or adapting my work patterns to facilitate me take the stress and strain away a little bit*. . . .*It felt overwhelming physically to clean the house*. *I just wanted to read a book or potter around*…*So I treated myself to a cleaner now which I wouldn’t have dreamt of doing before*, *but it’s changed my life*.*’* (Female, 50–69 years, PD)

Other patients described ways in which they would support themselves throughout difficult periods. At a basic level, this tended to involve patients trying to motivate themselves. When asked if she needed any emotional support, one woman said:

*‘…yes*, *but not very often*. *I sort of say to myself*, *look*, *get yourself out of this*, *you know*? *Pick yourself up*. *But I’ve always been rather independent*.*’* (Female, 70+ years, pre-dialysis)

However, many patients talked about hiding their emotional state from both healthcare staff and family members, and ‘bottling up’ their emotions. A number of participants described purposefully altering their behaviour and adopting a façade that made them seem more jovial or carefree–particularly when attending the kidney unit for in-centre dialysis sessions. One patient, when asked if staff on the kidney unit recognised when she was feeling down, replied:

*“Not really*, *no*, *because I’m usually bubbly when I go there*.*”* (Female, 70+ years, HD)

### Patient-staff interaction and kidney unit support

Participants often described a reluctance to talk to staff about emotional issues out of a fear that they may be perceived as complaining about their treatment. Male patients in particular often described talking about feelings as embarrassing:

*‘Well it’s a bit embarrassing to ask*, *to tell the nurses that your mental state isn’t very good*. *I think they should occasionally come and have a chat with you*…*Everyone’s in the same boat and you don’t feel like making yourself complain really*. *Seems that you’re complaining*.*’* (Male, 70+ years, HD)

Whilst some nurses and consultants on the kidney unit offered informal, ad hoc support during in-centre HD sessions or consultations, most patients felt that there was a lack of time on a busy kidney unit to discuss anything of substance beyond the immediate practicalities of clinical care. There was also a strong feeling that whilst staff members were well trained in delivering dialysis treatment and responding to medical issues, they were typically less able to address the emotional side of patients’ experience of ESKD:

*‘I don’t feel I could just phone them up and say “well I’m feeling really down today and feel really bad and had enough and I just want to give up the dialysis for good”*…*I just don’t feel like they’re there for that*…*They’ve not been trained for counselling and things like that*, *they’ve been trained for doing the dialysis*.*’* (Female, 50–69 years, HD)

Two of the kidney units included in this study had an on-site renal psychologist, yet few participants discussed sessions with psychologists or counsellors who had offered support. Where these sessions were mentioned, they were usually described as time-limited and did not seem to be routinely offered to participants who expressed feelings of distress. Conversely, several participants were highly complimentary about the support they had been offered by kidney unit staff. This was typically the case when patients had a long-standing and established relationship with particular staff who were adept at noticing changes in patient behaviour or demeanour which might indicate emotional issues:

‘*They know when I’m down in the dumps because I’m not bright and bubbly you know as I am*. *And they’ll say*. *There’s you know*, *you get one to one*…*They tell me everything and they don’t keep anything back from me*.*’* (Female, 70+ years, HD)

Linked to this, many patients described the improvements in wellbeing they had experienced from simply being able to talk to someone on the kidney unit about their distress, even if there were no immediate solutions that could be offered:

*‘They just sit there and listen*. *Even if there’s nothing they can say to help*, *it’s just like a listening ear*. *They’re just there listening*. *And if they’ve got something to say they give you input which half the time helps*.*’* (Female, under 50 years, HD)

### Impact of the treatment environment

An unexpected finding was the number of participants who described the impact of the treatment environment on the extent to which they felt able to raise emotional issues with staff. For some, this related to practical limitations posed by the kidney unit itself, such as a lack of private rooms in which patients could discuss emotional issues with staff members. Others talked about the staff deliberately attempting to create a light-hearted and jovial atmosphere on the unit which had mixed effects for patients. On one hand, participants frequently commended staff for this, arguing that an environment of relaxed informality fostered a sense of camaraderie amongst patients, which could alleviate some of the negative impacts of dialysis and reduce the potential dread that patients felt in relation to ESKD. However, for others, this was seen as creating an atmosphere in which people felt unable to discuss emotional problems for fear of bringing down the overall positivity of the unit. This led to a feeling for some that the kidney unit was not the appropriate place for conversations about emotional feelings to take place:

*“The choice would have been nice*. *I’m probably alright at the moment but I think you know there have been a few times in the past 12 months or so where I maybe could have done with just having a chat with somebody*.*”* (Male, 50–69, HD)

## Discussion

This study has shown that distress and emotional difficulties are commonly experienced by patients with ESKD, particularly with regard to the rigours of dialysis treatment and the restrictions that this places on patients’ daily lives. Participants described many problems and potential sources of distress, but their accounts were dominated by the difficulties they perceived in being able to cope with the often unanticipated impact of ESKD and its treatment, particularly in terms of the likelihood that they would experience emotional challenges. Patients who described feeling in control of their condition, and who had strong family and friend support networks reported being better able to manage their distress, and those who had long-standing positive relationships with staff on the kidney unit talked about the effective and welcome support they received.

However, many participants felt unable to raise emotional issues with healthcare staff, due to a perception that staff did not have the time to discuss non-medical issues, or that they lacked the relevant skills to handle any emotional issues raised. Added to this, the deliberately upbeat atmosphere on the kidney units–whilst positive and comforting for some–may have inadvertently created an environment in which patients felt it was inappropriate to talk about distress, and many participants described being embarrassed about addressing emotional issues and deliberately avoiding disclosing any information about their distress. Other research has found that for many patients with emotional difficulties, fear of the stigma of being perceived as suffering from a mental health issue is a significant barrier to sharing feelings with staff and/or taking up the offer of supportive interventions [28]. In the case of patients that have limited contact with kidney staff, such as those on home dialysis or transplant patients who may attend follow-up appointments relatively infrequently, the opportunities to raise emotional issues are correspondingly reduced and may add to patients’ reluctance to disclose distress to their healthcare professionals on the occasions that they do attend the kidney unit [29].

This study has a number of implications for how kidney units could improve the support offered to patients. Pre-dialysis education about both the physical and emotional impacts of treatment would help to manage patients’ expectations about what they may experience, and could go some way towards ‘normalising’ distress within ESKD and preparing patients for some of the potentially negative emotional consequences of treatment [30]. Helping patients to develop adjustment and coping mechanisms is also essential. This could be achieved through patient referral to renal psychology services, or by ensuring that healthcare staff are trained in the skills necessary to recognise and respond to distress in their patients. Existing models of adaptation to chronic illness give examples of factors that enable good or poor adjustment [31, 32]. Good adjustment may involve feeling a sense of control regarding disease management, acceptance of one’s illness, and high social support (cognitive factors) combined with problem-focused coping strategies, positive health behaviours and adherence to treatment (behavioural factors). In our data, the absence of explicit coping strategies was striking, and where coping strategies were being used at all, they tended to be passive strategies of suppressing distressing thoughts and feelings. This has been found in other studies of adjustment to chronic conditions [33, 34]. There have been positive reports about the benefits of peer support, in which shared experience has been found to help patients with Chronic Kidney Disease to come terms with their condition and develop appropriate coping mechanisms for distress. Thus, the encouragement of such mutual support amongst patients with ESKD may be beneficial [35].

In terms of how patients can be guided about coping with distress, there seems to be a missing link between patient needs and staff behaviour [30]. Staff may avoid talking about distress because they are unsure of how to help and may feel unable to resolve patients’ emotional issues. The offer of formal psychological support may benefit some patients, however, our data showed that patients may simply want to talk to a sympathetic listener rather than necessarily reaching a solution to their problems. Kidney services should work to remove the barriers to patients feeling able to disclose emotional issues, and the mutual recognition that such issues are normal and to be expected within a chronic condition like ESKD is essential. This could be aided by open and transparent communication between healthcare professionals and patients at all stages of ESKD, so that expectations about the likelihood of distress could be managed, and challenges in dealing with RRT, along with strategies to help minimise problems, could be discussed with patients and their caregivers.

### Limitations

Although our study sample was large, and drawn from four diverse hospital Trusts in the West Midlands, the extent to which our findings may be generalisable could be limited. There is also a possibility that the patients who were interviewed face-to-face may have expressed substantially different opinions compared with those who were interviewed over the telephone. However, the extent to which this may have biased our findings is likely to be minimal given the very small numbers involved (three face-to-face interviews compared with 43 telephone interviews).

## Conclusions

Despite UK national policy and guidelines which mandate the integration of emotional and psychological health into routine care for patients with chronic conditions, this study suggests that there is still progress to be made in the case of chronic kidney disease. Patient distress was substantial and few patients were able to develop appropriate coping strategies to manage their emotional issues. Instances in which kidney unit staff had offered effective emotional support to patients were rarely raised by participants, and those that were described were largely due to the input of particular staff with whom patients had long-standing relationships rather than this support being routinely offered by kidney units. The findings of this study are likely to be relevant to other long-term conditions where levels of distress are known to be high.
